# Neighborhood educational disparities in active commuting among women: the effect of distance between the place of residence and the place of work/study (an ACTI-Cités study)

**DOI:** 10.1186/s12889-017-4464-8

**Published:** 2017-06-12

**Authors:** Camille Perchoux, Julie-Anne Nazare, Tarik Benmarhnia, Paul Salze, Thierry Feuillet, Serge Hercberg, Franck Hess, Mehdi Menai, Christiane Weber, Hélène Charreire, Christophe Enaux, Jean-Michel Oppert, Chantal Simon

**Affiliations:** 1 CRNH Rhône-Alpes, Pierre Benite, France; 2 CARMEN INSERM U060/University of Lyon1/INRA U1235, Oullins, France; 3 CENS, Hospices Civils de Lyon, Pierre Benite, France; 4 Department of Family Medicine and Public Health & Scripps Institution of Oceanography, University of California, 9500 Gilman Drive, San Diego, La Jolla, 92093 CA USA; 5 Paris-Est University, Labex Futurs Urbains, Marne-la-Vallée, France; 6 Department of Geography, LADYSS, Paris 8 University, Paris, France; 7 Université Paris 13, Sorbonne Paris Cité - EREN (Equipe de Recherche en Epidémiologie Nutritionnelle), U1153 Inserm, Inra, Cnam, Centre de Recherche en Epidémiologie et Biostatistiques; CRNH IdF, Bobigny, France; 8 Laboratoire Image, Ville et Environnement, Université de Strasbourg, Strasbourg, France; 9 Paris-Est University, Department of Geography, Lab-Urba, Créteil, France; 10 Université Pierre et Marie Curie-Paris 6, Dept of Nutrition Pitié-Salpêtrière Hospital (AP-HP), Institute of Cardiometabolism and Nutrition (ICAN), Paris, France; 11 Service d’Endocrinologie, Diabète, Nutrition Centre Hospitalier Lyon Sud, 165 Chemin du Grand Revoyet, F69310 Pierre-Bénite, France

**Keywords:** Active commuting, Neighborhood education, Distance to work, Effect measure modification, Social environment

## Abstract

**Background:**

Active transportation has been associated with favorable health outcomes. Previous research highlighted the influence of neighborhood educational level on active transportation. However, little is known regarding the effect of commuting distance on social disparities in active commuting. In this regard, women have been poorly studied. The objective of this paper was to evaluate the relationship between neighborhood educational level and active commuting, and to assess whether the commuting distance modifies this relationship in adult women.

**Methods:**

This cross-sectional study is based on a subsample of women from the Nutrinet-Santé web-cohort (*N* = 1169). Binomial, log-binomial and negative binomial regressions were used to assess the associations between neighborhood education level and (i) the likelihood of reporting any active commuting time, and (ii) the share of commuting time made by active transportation modes. Potential effect measure modification of distance to work on the previous associations was assessed both on the additive and the multiplicative scales.

**Results:**

Neighborhood education level was positively associated with the probability of reporting any active commuting time (relative risk = 1.774; *p* < 0.05) and the share of commuting time spent active (relative risk = 1.423; *p* < 0.05). The impact of neighborhood education was greater at long distances to work for both outcomes.

**Conclusions:**

Our results suggest that neighborhood educational disparities in active commuting tend to increase with commuting distance among women. Further research is needed to provide geographically driven guidance for health promotion intervention aiming at reducing disparities in active transportation among socioeconomic groups.

**Electronic supplementary material:**

The online version of this article (doi:10.1186/s12889-017-4464-8) contains supplementary material, which is available to authorized users.

## Background

The benefits of walking and cycling for transportation on many health outcomes (i.e. cardiovascular diseases, colon cancer, mortality) are widely recognized [1–6] and the promotion of active commuting has been suggested as an effective way to increase levels of habitual physical activity [7, 8], especially among women [7]. Previous ecological studies highlighted the importance of social correlates and/or determinants of walking and cycling [9]. Indeed some studies have observed social disparities in physical activity either at the individual [10–14] or at the neighborhood level [11, 15–18]. Social neighborhood factors do matter for health, and among them a high-education neighborhood is one criteria of a “high-opportunity neighborhood” as defined by the Moving To Opportunity program [19]. More specifically, the mechanism through which neighborhood educational level influences physical activity has been hypothesized to correspond to “a high average education in the neighborhood may stimulate values that are favorable to a healthy and physically active lifestyle” ([18], p. 5). As such, neighborhood-education inequality has been associated with smoking [20] and alcohol consumption [20, 21], while low-education neighborhood has been associated with weight-related outcomes [22–25]. Neighborhood educational disparities in non-motorized traffic trail [26, 27], overall walking [15], walking for transportation [17] and walking for recreation [16, 18] have been reported in adult populations. Therefore, disparities in neighborhood educational level might turn into neighborhood educational disparities in active commuting, supporting “low-active low-educated neighborhood” as priority target for community-based interventions promoting physical activity [28]. However to our knowledge, except one study [17], no attention has been paid to the effect of neighborhood educational level on active transportation. It appears therefore relevant to clarify whether educational disparities in active commuting occur at the neighborhood level, independently of individual level of education. In this regard, as women have been shown to be more sensitive to the effect of the effect of socio-economic deprivation on physical activity than men [29], and poorly studied, this studies focused exclusively on women.

Distance between home and place of work/study has been consistently identified as a major determinant/correlate of active commuting [30]. Although the World Health Organization considered that 5 km remains a feasible distance to engage in transportation physical activity among adults ([31] cited in [32]), commuting distance appears as a strong barrier to active commute (to/from place of work/study) [30, 32–38]. However, few authors suggested that commuting distance (i.e. distance to/from place of work/study) might modify the association between environmental exposure and active commuting [39]. Since perceived and objective distance are identified as a strong barrier to active commuting and that walking or cycling a long way may tire individuals [40], the influence of a favorable educational environment for active commuting might provide a substantial support for the adoption or the maintenance of an active lifestyle. Alternatively, we hypothesized that individuals living in a less educated area far from their place of work/study might suffer from a double burden of distance and lack of supportive values/context for active commuting. To our knowledge, few studies examined the potential effect measure modification of travel distance on the association between active commuting and environmental exposure among children only, and presented mixed results [41, 42].

In addition, active commuting has been systematically examined measuring as the time spent in active commuting to evaluate whether specific populations meet the physical activity guidelines [43]. However, some authors argued in favor of a wider diversity of active commuting measurements to provide further guidance for sustainable urban planning and population health interventions [44, 45]; e.g. (changes in) commute mode share, number of commuting trips [44]. Since commuting to place of work/study might imply multimodal transportation modes (i.e. transit trip), including both active and sedentary travel modes [46], we advocate for a comprehensive measure of active commuting that considers the active commuting time in relation with the whole travel time and related transportations modes.

The first objective of this study was to assess specifically in women the association between neighborhood education level and both (i) the likelihood of reporting any active commuting time, and (ii) the share of total commuting time made by active transportation modes. The second objective was to evaluate whether distance to place of work/study modifies the relationship between neighborhood education and active commuting. We hypothesized that a positive association between neighborhood education and active commuting would be stronger with increasing distance to place of work/study. In other words, among individuals living far from their place of work/study, we expected the neighborhood education level to be a stronger correlate of engaging in active commuting, compared to individuals living close to their place of work/study.

## Methods

### The nutrinet-santé study

The Nutrinet-Santé Study is a web-based prospective observational cohort established in France in May 2009 to investigate the relationships between nutrition and health. Volunteer participants from the general population, aged 18 y or older, living in France and having access to the Internet fill in self-administered web-based questionnaires at baseline and then regularly during follow-up using a dedicated, secure website (for further details regarding the Nutrinet-Santé Study, see www.etude-nutrinet-sante.fr). The study was approved by the “Comité National Informatique et Liberté” (CNIL n°908,450, n° 909,216 and DR-2012-576) and the Institutional Review Board of the French Institute for Health and Medical Research (IRB Inserm n°0000388FWA00005831). Informed consent was obtained from all individual participants included in the study. A detailed description of the design of the Nutrinet-Santé Study is available elsewhere [47].

### Study design and population of the ACTI-Cités study

This cross-sectional study is based on a subsample of the Nutrinet-Santé study, including working or studying women only, who lived in the Bas-Rhin and Rhône *départements* - two comparable French *départements* -, and who completed the “Sedentary, Transportation and Activity Questionnaire” (STAQ) administered from February to August 2013 (completion rate was 48.5%). An automated e-mail informed participants of the necessity to complete their profiles by filling out this questionnaire (which took less than 20 min on average) in their personal space on the website of the NutriNet-Santé cohort study. Participants had previously completed baseline questionnaires on health, lifestyle and socio-demographic factors at inclusion.

From the initial sample of 1973 women living in the Rhône and Bas-Rhin “départements” who completed the STAQ and declared no physical motor injuries [48], we excluded 627 participants who declared no employment or study activity, 92 participants who declared no commuting time (i.e. working at home), 7 participants for missing socio-demographic data and 31 participants for missing place of work/study location. 47 participants were further excluded for declaring a place of work/study more than 100 km distant from their home, since our study focus was on short to moderate commuting distance [49]. The final study sample included 1169 female participants.

### Measures

#### Active commuting

The time of active commute was self-reported by participants. Participants were asked to report the following information: i) in the last 4 weeks, how many times a week did you travel from home to your main work? (Count the outward journeys only); ii) how many days per week, during the last 4 weeks, you have used each form of transport to go (and/or come back) from your work (i.e. car/motor vehicle, public transport, walk, cycle, other active transport (skate, scooters, rollers…)); iii) the average length of time per day, during the last 4 weeks, you have spent using each form of transport to go (and/or come back) from your work. For each transportation mode, durations of commute (hrs/week) were computed. Active commuting duration was calculated by summing the time spent walking, cycling, and using other active transportation modes. As such, walking or cycling transit trips of short durations (i.e. walking or cycling to the station, or from the station to the place of work) were included in the active commuting duration. Total travel duration was calculated by summing all reported durations of commute. We then calculated the percentage of total active commuting time by dividing the active commuting duration by the total travel duration. This variable was defined in the whole population (*N* = 1169). We then created a count variable among active commuters (*N* = 537) representing 10 categories in the percentage of active commuting (1 = [>0% - 10%]; 10 = [>90% - 100%]), reflecting the probability of increasing the share of active commuting by 10%. The distribution of this outcome variable is provided in Additional file 1.

Finally, two distinct dependent variables were considered: (i) a binary variable indicating the reporting of any active commuting (*N* = 1169), and (ii) a count variable defined among active commuters (*N* = 537) indicating the share of total commuting time spent active.

#### Neighborhood education level

The neighborhood education level was defined as the proportion of residents with university education diploma as obtained from the 2010 population census at the French census block level (i.e. IRIS - a French acronym for “blocks for incorporating statistical information”) designed by the National Census Bureau Institut (INSEE – Institut national de la statistique et des études économiques). First, the neighborhood educational level data were disaggregated in a Geographic information system (GIS) grid units of 200 m × 200 m [50]. In a second step, values of neighborhood education levels at GIS grid level were computed within a 1 km circular buffer radius around each participant’s residence, and then divided into quartiles (quartiles’ threshold values: 25, 32, 42%) corresponding to high, middle-high, middle-low and low levels.

#### Assessment of residence and workplace or study location and commuting distance

Participants were asked to report the location of their place of residence, geolocated at the address, and the location of their place of work/study at the municipality or the “arrondissement” unit. The commuting distance (distance to place of work/study) was then defined as the Euclidian distance between the place of residence and the centroïd of the municipality or “arrondissement” unit (city sub-unit) where participants indicated they were working/studying [51] (quartiles’ threshold values: 2.76 km, 5.97 km, 13.59 km), using ArcInfo 9.3 (ESRI Inc., Redlands, CA, USA).

#### Individual covariates

The following characteristics were considered for adjustment: age, individual education (4 categories: no education and primary education, secondary education, lower tertiary education, and upper tertiary education), having at least one child under the age of thirteen at home, and the “département” of residence (Bas-Rhin “département” and Rhône “département”).

### Analyses

Variation and trends across neighborhood education categories and commuting distance in (i) the reporting of active commuting time or not and (ii) the share of total commuting time spent active were assessed using descriptive statistics and Jonckheeree-Terpstra (JT) test; *p*-values are reported.

The association between neighborhood education and active commuting was estimated using two separate regression models. The probability of reporting any active commuting was fitted using log-binomial regression models, with effect measures interpreted as relative risks (RR) and binomial regressions, with effect measures interpreted as risk differences (RD) [52]. As recommended elsewhere, when log-binomial and binomial regression models did not converge, we used Poisson regression with robust error variance with log and identity links, respectively [53]. Due to the over-dispersion of the count variable (i.e. share of total commuting time spent active), negative binomial regressions were fitted to evaluate the share of total commuting time spent active among active commuters. This model represents the probability of increasing the share of total commuting time spent active by 10%. Additional analyses were computed by using the absolute time spent in active commuting as outcome, in order to evaluate whether neighborhood education level was consistently associated with the share and the absolute time spent in active commuting.

Each covariate included in the model was tested first using bivariate analyses to evaluate potential confounding [54], and all the tested covariates were significantly associated with active commuting and neighborhood education level and therefore considered as confounders. No confounding factors regarding the relationship between distance to work and active commuting were considered since our main aim was to evaluate whether the neighborhood education level association vary across distance strata, interpreted as measure modification [55].

Then, we estimated multiplicative interactions by including an interaction product term in both log-binomial, and negative binomial models respectively (Neighborhood education level x Distance) [56]. In order to assess the distance threshold at which the effect measure modification was stronger, we repeated the analyses on the multiplicative scale using increasing distance threshold by 500 m from 1000 m to 6000 m. We then selected the distance at which the effect of the interaction rate ratio (IRR) (which represents the exponential form of the interaction product term) was stronger to performed stratified analyses.

Finally, we evaluated the heterogeneity of the neighborhood education association across distance to work stratum by performing Cochran’s Q test [57], for both relative risks (RR) and absolute risks (RD). We determined that a Cochran’s Q test with a *p*-value <0.10 indicates that the two differing stratum-specific estimates are derived from two distinct distributions, while the null hypothesis (*p* > 0.10) indicates that the two stratum estimates are part of the same distribution and differences in estimates are only due to ‘sampling variability’ [57].

Sensitivity analysis assessed the impact of using another definition of commuting distance on the probability of reporting any active commuting and the share of total commuting time spent active. Sensitivity analysis assessed the impact of using another definition of commuting distance on the probability of reporting any active commuting and the share of total commuting time spent active. Distances were estimated using the types of transportation modes and the commuting duration by transportation modes reported by the participants. We applied the following travel speed for each transportation mode: 23 km/h for car, 12 km/h for public transport, 12 km/h for cycling, 4 km/h for walking, 10 km/h for other active travel modes of transportation [58]. By multiplying the travel speed by the commuting time, we were able to approximate a commuting distance. All analyses were performed using SAS 9.3 (SAS Institute, Inc., Cary, North Carolina).

## Results

Descriptive information of this final sample is provided in Table 1.

**Table 1:** Descriptive information on the sample used in the study, *N* = 1169

Variables	% or mean	N or SD
Individual variables		
Age (mean, years)	41,88	10.98
Living with a child under the age of 13 y (%)	33.18	388
Individual education (%)		
High	42.26	494
Middle-high	34.90	408
Middle-low	16.85	197
Low	5.99	70
“Département” of residence (%)		
Bas-Rhin “département”	30.54	357
Rhône “département”	69.46	812
Neighborhood social variable		
Neighborhood education (%)		
High	24.64	288
Middle-high	23.61	276
Middle-low	27.54	322
Low	24.21	283

**Fig. 1:**
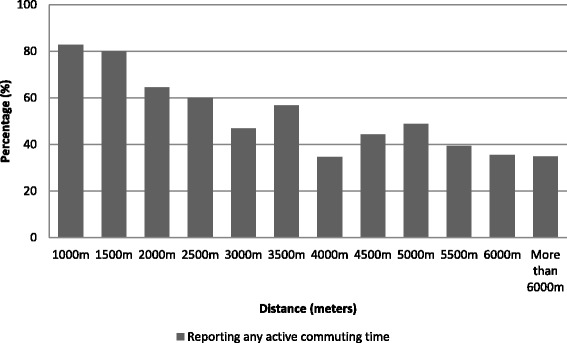
Reporting any active commuting time by commuting distance to place of work/study (*N*=1169)

**Fig. 2:**
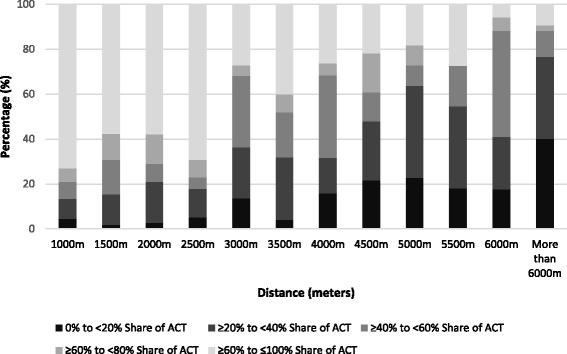
Share of total commuting time spent active among active commuters by distance to place of work/study (*N*=537). ACT: Active commuting time

54.1% of the sample declared no active commuting time. Overall, the mean time spent commuting per week was 3.33 h (interquartile range: 3.42). The reporting of any active commuting time showed a positive trend (JT test, *p*-value <0.001) with neighborhood education level. As observed in Fig. 1, the reporting of any active commuting time decreased with increasing commuting distance (JT test, *p*-value <0.001). Among active commuters, the mean time per week spent in active commuting was 1.67 h (interquartile range: 1.83). Descriptive analyses showed a negative trend (JT test, *p*-value <0.001) between the share of total commuting time spent active and the commuting distance (Fig. 2). The share of total commuting time spent active increased with neighborhood education level (JT test, *p*-value <0.001).

### Associations between neighborhood education level and active commuting

The probabilities of reporting any active commuting time and the share of total commuting time spent active among active commuters according to neighborhood education level are presented in Tables 2 and 3 respectively, in terms of relative risks and risk differences.

**Table 2:** Association between neighborhood education level and the probability of reporting any active commuting, *N* = 1169^a^

	RR	95% CI	RD	95% CI
Neighborhood education (vs. low)				
High	1.77	(1.48, 2.13)	0.29	(0.21, 0.37)
Middle high	1.27	(1.04, 1.55)	0.09	(0.01, 0.18)
Middle low	0.93	(0.75, 1.15)	−0.02	(−0.10, 0.54)

*RR* relative risk, *RD* risk difference, *CI* confidence interval

^a^Log-binomial and binomial regression models adjusted for age at the mean, low individual education, living with a child under the age of thirteen, and living in the Rhône “département”

**Table 3:** Association between neighborhood level education and the share of total commuting time spent active among active commuters, *N* = 537^a^

	RR	95% CI	RD	95% CI
Neighborhood education (vs. low)				
High	1.42	(1.24, 1.65)	0.36	(0.21, 0.50)
Middle high	1.23	(1.05, 1.43)	0.20	(0.05, 0.36)
Middle low	1.04	(0.89, 1.22)	0.04	(−0.12, 0.20)

*RR* relative risk, *RD* risk difference, *CI* confidence interval

^a^Negative binomial regression models adjusted for age at the mean, low individual education, living with a child under the age of thirteen, and living in the Rhône “département”

Living in a highly educated neighborhood increased the risk of reporting any active commuting time by 77% on the relative scale (RR) compared to participants living in a low educated neighborhood. On the absolute risk difference scale, the RD for high educated neighborhood vs. low educated neighborhood was 0.29 (95% CI: 0.21, 0.37). Similarly, among active commuters, residing in a highly educated neighborhood was associated with a 42% increase in the share of total commuting time spent active on the relative scale. On the absolute risk difference scale, the RD for high educated neighborhood vs. low educated neighborhood was 0.36 (95% CI: 0.21, 0.50).

Additional analyses showed a positive association between neighborhood education level and the absolute time spent active commuting both on the relative risk scale and the absolute risk difference scale (Additional file 2).

### Neighborhood education level, distance to work and likelihood of reporting any active commuting

**Fig. 3:**
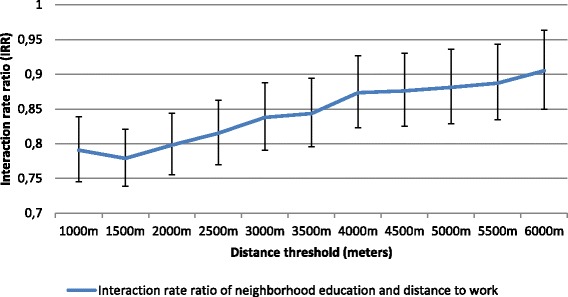
Analyses of distance threshold effect and neighborhood education level on the probability of reporting any active commuting (*N*=1169)

After accounting for distances to work independent effect, high neighborhood education level (vs. low) was still associated with the risk of reporting any active commuting (Table 4, Model 1). The interaction model indicated a negative interaction between commuting distance (1 km increase) and neighborhood education in their association with the risk of reporting any active commuting time (Table 4, Model 2). Sensitivity analyses using another definition of distance to work/study (i.e. based on travel speed) confirmed these results (Additional file 3). Repeated interaction analyses on the multiplicative scale with varying distance thresholds indicated that the modification effect (using IRRs) of distance to work on the relationship between neighborhood education and risk of reporting any active commuting was greater at 1500 m (Fig. 3).

**Table 4:** Association between neighborhood education, distance to work and the probability of reporting any active commuting, *N* = 1169

Main models				
Regression coefficients	Models 1^a^		Models 2^b^	
	β	95% CI	β	95% CI
Neighborhood education				
High	0.52	(0.34, 0.70)	0.73	(0.49, 0.98)
Middle high	0.19	(−0.01, 0.39)	0.45	(0.18, 0.73)
Middle low	−0.09	(−0.30, 0.12)	0.22	(−0.07, 0.51)
Low	Ref.		Ref.	
Distance to work				
1 km increase in distance	−0.01	(−0.02, 0.00)	0.01	(0.006, 0.02)
Neighborhood education and commuting distance				
High x Distance	-		−0.03	(−0.05, −0.01)
Middle high x Distance	-		−0.03	(−0.05, −0.01)
Middle low x Distance	-		−0.02	(−0.04, 0.00)
Low x Distance	-		Ref.	
*p*-value for interaction			< 0.001	
Stratified analyses				
Estimated effect measures	RR	95% CI	RD	95% CI
Neighborhood education at short distance to place of work/study (< 1500 m), *N* = 146				
High	1.06	(0.87, 1.29)	0.08	(−0.10, 0.26)
Middle high	0.87	(0.69, 1.09)	−0.10	(−0.31, 0.10)
Middle low	0.71	(0.50, 1.02)	−0.22	(−0.49, 0.04)
Low	Ref.		Ref.	
Neighborhood education at long distance to place of work/study (> 1500 m), *N* = 1023				
High	1.77	(1.44, 2.20)	0.27	(0.18, 0.36)
Middle high	1.24	(0.98, 1.56)	0.08	(−0.01, 0.17)
Middle low	0.97	(0.77, 1.23)	0.00	(−0.08, 0.08)
Low	Ref.		Ref.	
*p*-value for Cochran’s Q test	< 0.001		0.036	

For stratified analyses, log-binomial regression model did not converge at the “short distances” level; we therefore performed Poisson regression models with robust error variance for both levels

*RR* relative risk, *RD* risk difference, *CI* confidence interval

^a^Log binomial regression model 1 included neighborhood education levels, distance to work, and was adjusted for age at the mean, low individual education, living with a child under the age of thirteen, and living in the Rhône “département”

^b^Log binomial regression model 2 included neighborhood education level, distance to work, the interaction term between neighborhood education levels and distance to work, and was adjusted for age at the mean, low individual education, living with a child under the age of thirteen, and living in the Rhône “département”

Stratified analyses showed a stronger association between neighborhood education level and likelihood of reporting any active commuting at distance to work longer than 1500 m in comparison to distance to work shorter than 1500 m. For instance, the increased relative risk of high neighborhood education at long distance to work (>1500 m) was 77% (RR = 1.77; 95% CI: 1.44, 2.20), versus 6% (RR = 1.06; 95% CI: 0.87, 1.29) at short distance to work (≤1500 m). Similarly, the absolute risk of residing in a highly educated neighborhood (in comparison to low neighborhood education group) was greater at long distance (RD = 0.27; 95% CI: 0.18, 0.36) versus short distance to work (RD = 0.08; 95% CI: (−0.10, 0.26). Cochran’s Q tests were significant on both relative (*p* < 0.001) and absolute (*p* < 0.05) scales.

### Neighborhood education level, distance to work and share of total commuting time spent active among active commuters

**Fig. 4:**
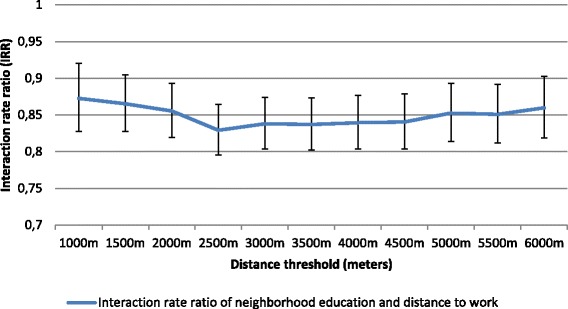
Analyses of distance threshold effect and neighborhood education level on the share of total commuting time spent active among active commuters (*N*=537)

As shown in Table 5 (Model 1), after accounting for distances to work independent effect, neighborhood education levels were no more associated with the share of total commuting time spent active for all neighborhood education levels. Interaction analyses showed no significant modification effect of distance to work/study (1 km increase) on the relationship between neighborhood education level and share of total commuting time spent active. Sensitivity analyses confirmed the absence of interaction with distance to place of work/study considered as a continuous variable (Additional file 4). Yet repeated analyses (using IRRs) indicated a greater modification effect at 2500 m (Fig. 4).

**Table 5:** Association between neighborhood education, distance to work and share of total commuting time spent active among active commuters, *N* = 537

Main models				
Regression coefficients	Model 1^a^		Model 2^b^	
	β	95% CI	β	95% CI
Neighborhood education				
High	0.11	(−0.03, 0.25)	0.21	(0.04, 0.38)
Middle high	0.00	(−0.14, 0.15)	0.10	(−0.08, 0.28)
Middle low	−0.11	(−0.25, 0.04)	0.05	(−0.15, 0.25)
Low	Ref.		Ref.	
Distance to work				
1 km increase in distance	−0.02	(−0.03, − 0.02)	−0.02	(−0.02, −0.01)
Neighborhood education and Distance to work				
High x Distance	-		−0.01	(−0.03, 0.00)
Middle high x Distance	-		−0.01	(−0.02, −0.01)
Middle low x Distance	-		−0.02	(−0.03–0.00)
Low x Distance	-		Ref.	
*p*-value for interaction			0.20	
Stratified analyses				
Estimated effect measures	RR	95% CI	RD	95% CI
Effect of neighborhood education at short distance to work (<2500 m), *N* = 196				
High	1.04	(0.88, 1.22)	0.03	(−0.13, 0.20)
Middle high	1.02	(0.85, 1.22)	0.02	(−0.16, 0.20)
Middle low	1.04	(0.85, 1.27)	0.04	(−0.16, 0.24)
Low	Ref.		Ref.	
Neighborhood education at long distance from work (>2500 m), *N* = 341				
High	1.29	(1.07, 1.56)	0.26	(0.07, 0.45)
Middle high	1.17	(0.96, 1.41)	0.15	(−0.04, 0.34)
Middle low	1.02	(0.85, 1.23)	0.02	(−0.17, 0.21)
Low	Ref.		Ref.	
*p*-value for Cochran’s Q test	0.080		0.082	

*RR* relative risk, *RD* risk difference, *CI* confidence interval

^a^Negative binomial regression model 1 included neighborhood education levels, distance to work, and was adjusted for age at the mean, low individual education, living with a child under the age of thirteen, and living in the Rhône “département”

^b^Negative binomial regression model 2 included neighborhood education level, distance to work, the interaction term between neighborhood education levels and distance to work, and was adjusted for age at the mean, low individual education, living with a child under the age of thirteen, and living in the Rhône “département”

In stratified analyses, the association between neighborhood education level (high vs. low) and the share of total commuting time spent active becomes significant at long distance to place of work/study on both the relative and the absolute scales. More specifically, stratified analyses indicated a stronger association between high neighborhood education (vs. low) and share of total commuting time spent active at long distance (>2500 m) versus short distance to work (≤2500 m), on both the relative risk scale and the absolute risk scale (Table 5). Cochran’s Q tests were significant at α =0.1 on the relative scale (*p* = 0.080) and on the absolute scale (*p* = 0.082), indicating a heterogeneous effect of neighborhood education on share of total commuting time spent active by distance to work strata.

## Discussion

### Principal findings

In this study, neighborhood education level was positively associated with active commuting to place of work/study and distance commuted modified this association with stronger neighborhood association for distance traveled up to 1500 or 2500 m.

### Neighborhood educational disparities on active commuting

In this study, neighborhood education level was examined based on the hypothesis that it is a proxy for a safe and “trusting” environment and social norms favorable to physical activity, in line with previous research [11, 15–18]. Both the reporting of active commuting time and share of total commuting time spent active were associated with residence neighborhood educational level, independently of individual education. Similarly, a French cohort based in the Ile-de-France area reported a positive association between neighborhood educational level and both recreational physical activity [16, 18] and active transportation [17]. Also, Ross (2000) observed in Illinois in the US that individuals living in highly educated neighborhoods were more likely to walk than their counterparts living in less educated neighborhoods [15]. The authors suggested that since walking was a visible outdoor activity, it might encourage mimetic behaviors and create a culture of walking within the neighborhood. The association between neighborhood education level and the absolute time of active commuting tend to confirm this hypothesis. However, except from a few studies [44, 46, 59, 60], multimodal transport behavior remains an under-explored area in place and health research. The ‘mechanisms of influence’ of neighborhood education on active commuting to work would need to be further investigated, considering the potential mediator effects of personal (socio-demographic characteristics, personal representation of transportation modes), interpersonal (e.g. social support) and environmental (e.g. residential density, traffic safety, neighborhood aesthetics, greenness, etc.) factors [11].

### The modifying effect of distance to place of work/study

A key finding of this study is the modifying effect of distance traveled on the association between neighborhood education level and active commuting. The results support our hypothesis that there would be a stronger neighborhood educational association for longer distances on the reporting of active commuting. Evidence is less clear regarding the share of total commuting time spent active among active commuters since the interaction was not significant with distance as a continuous variable (1 km increase). In the analyses, the homogeneity tests were conducted using Type I error with α level set as 0.10. This methodological choice was made to increase the probability of detecting a modifying effect of distance on the relationship between neighborhood education and the share of total commuting time spent active among active commuters. Indeed, given the low statistical power of homogeneity tests and the small sample size involved for this outcome (*N* = 537), a more conservative choice of *p*-value for significance (α = 0.05) would have reduced the probability to detect “real” heterogeneity [57, 61, 62].

In the literature, there are few data on the analysis of modifying effect of distance on the relationship between neighborhood factors and active commuting, and results are mixed. In one study from Panter and colleagues among children in the county of Norfolk, the authors found no modifying effect of distance on the relationship between residential neighborhood socioeconomic deprivation and active commuting [41]. However, in another study [42], the same authors found a modifying effect of distance whereby parental attitudes towards their child were more influential on short distances (e.g. 0 to 1 km) while safety was stronger associated on long distances (e.g. more than 2 km). It should be noticed that the distance may not have the same impact on active transportation among children and adults.

Another important result concerns the varying distance threshold at which the effect measure modification was stronger, namely at 1500 m for the reporting of any active commuting and, at 2500 m for the share of total commuting time spent active. We hypothesized that the variation of distance threshold between the two outcomes might relate to a different degree between individuals of perception of distance as a barrier to active commuting [63, 64]. Indeed, in the whole sample encompassing both active and passive commuters, distance greater than 1500 m from home might not represent a feasible distance by walk or cycle, while among participants already experiencing active commuting, the barrier effect of distance is weaker and appears on longer distance to travel (i.e. 2500 m). This hypothesis that the barrier effect of distance depends on the level of individuals’ physical activity is along the lines of a study from Wuerzer et al. [63] based on a younger age category, and indicating that “students who cycle for transportation are more likely to cycle regardless of distance” (p. 102).

### Limitations and strengths

One key strength of this study relies on the use of specific questions on transport behaviors, detailing the times spent on each active vs. non active transportation modes per week. Moreover, this is one of the first study providing a deep investigation of the modification effect of distance to place of work/study - including distance thresholds - on the association between the neighborhood social environment and active commuting. Nevertheless, one major limitation of this study relies on its cross-sectional design that prevents to consider residential self-selection [65], and to draw causal inferences regarding the effect of neighborhood education level on active commuting. The use of a unique neighborhood socio-economic indicator (neighborhood education level) prevent to generalize on our results to neighborhood social disparities, especially since neighborhood income and neighborhood education might have opposite influence on physical activity outcomes [15, 66]. As such, Hankey et al. observed a positive association between neighborhood education and both levels of pedestrian and bicycle traffic, but a negative association between neighborhood household income and bicycle traffic [27]. Furthermore, the Nutrinet-Santé Cohort include proportionally more highly-educated highly individuals compared to the National Census data [67]. However, despite the high-education level of the participants, we observed neighborhood educational disparities in active commuting to work/study, suggesting that if it has a potential impact, it might have underestimated such disparities. Given the relatively small sample of participants involved in the evaluation of an effect heterogeneity of distance to work/study on the share of total commuting time spent active (*N* = 537), replication studies on other populations and larger sample sizes are needed to assess the consistency of the results. Another source of uncertainty pertains to our definition of distance to place of work/study. The accuracy of the distance estimates is moderate as it does not account for the street network (data not available), and the place of work/study was geolocated at the municipality or arrondissement level. However, using an Euclidian distance compared to a street network distance might only have a low impact since a study based in the Bas-Rhin department evaluated the correlation between street-network distance and Euclidian distance and observed a very strong correlation (above 0.97) [68]. Measurement errors in the definition of the distance might have been introduced since the workplace was geocoded at the centroid of the municipality/arrondissement. We believe that this measurement error is not differential since it affects the whole sample, and the distance between the centroid of the municipality and the place of work is unlikely to be correlated with the education level of the neighborhood, or the active commuting behavior. Sensitivity analyses based on another definition of commuting distance provided similar results. Another limitation includes the use of self-reported measures of commuting time which are frequently under/overestimated [69]. In addition, the assessment of neighborhood education level is home-centered, which might have introduced measurement errors in exposure definition as it does not account for the neighborhood education level at the workplace nor along the route between the two activity locations (i.e. home, place of work/study) [70]. Yet, a study in the Ile-de-France Region (France) found an association between workplace neighborhood education and walking to work [17]. As this study focused exclusively on women, results cannot be generalized to men.

### Implications for public health and opened questions

This study highlights the complexity of the association between neighborhood educational level and active commuting. More specifically, it suggests that the strength and the significance of the association between neighborhood education level and active commuting would be distance-specific among women.

In terms of analysis strategy for studies examining environmental influences on active commuting, further attention should be paid to the potential modifying effect of commuting distance on the influence of either social or physical environmental factors. Replication analyses are also needed to evaluate the share of total commuting time made by walking and cycling, separately. Indeed, the strength of the association between active commuting and distance to work may differ between walking and cycling [39], as some authors observed a stronger influence of distance on walking than cycling [71]. Such additional analyses would have been underpowered in our study, given the relatively small baseline sample size of active commuters, and the low statistical power of homogeneity tests [57].

In terms of population health interventions, our findings suggest that community-based interventions designed to promote active commuting among women living in educationally disadvantaged neighborhoods [29] would be most important for individuals living far from their place of work. Subject to causal inference (not evaluated in this cross-sectional study), our results encourage interventions in low educated neighborhoods by targeting in priority residential areas located at more than 1500 m from potential sources of employment, considering that distance to work is unlikely to be easily modified by intervention [33].

## Conclusion

In summary, neighborhood education level is positively associated in working women with the probability of reporting any active commuting time and the share of commuting time spent active. Commuting distance to place of work/study modifies the relationship between neighborhood education level and active commuting; more specifically the effect of neighborhood education is greater at long distance to work/study. While the results are robust, replication studies are required given the lack of evidence regarding distance as a modifier of social environment effect on active transportation.

## Additional files

Additional file 1: Distribution of the “share of total commuting time spent active” among active commuters, *N* = 537. (DOCX 13 kb)

Additional file 2: Additional analysis: association between neighborhood education level and the absolute time of active commuting, *N* = 537^a^. (DOCX 12 kb)

Additional file 3: Sensitivity analysis: association between neighborhood education, distance to work* and the probability of reporting any active commuting (*N* = 1169). (DOCX 14 kb)

Additional file 4: Sensitivity analysis: association between neighborhood education, distance to work* and share of total commuting time spent active among active commuters (*N* = 537). (DOCX 13 kb)
