# Supplementary material for: Neighborhood educational disparities in active commuting among women: the effect of distance between the place of residence and the place of work/study (an ACTI-Cités study)
Source: BMC Public Health. 2017 Jun 12;17:569. doi: 10.1186/s12889-017-4464-8 (PMC5469012; doi:10.1186/s12889-017-4464-8)
Supplement: Supplementary file 1 — Distribution of the “share of total commuting time spent active” among active commuters, N = 537. (DOCX 13 kb) [file 12889_2017_4464_MOESM1_ESM.docx]

**Additional file 1. Distribution of the “share of total commuting time spent active” among active commuters, N=537.**
