# Supplementary material for: Neighborhood educational disparities in active commuting among women: the effect of distance between the place of residence and the place of work/study (an ACTI-Cités study)
Source: BMC Public Health. 2017 Jun 12;17:569. doi: 10.1186/s12889-017-4464-8 (PMC5469012; doi:10.1186/s12889-017-4464-8)
Supplement: Supplementary file 2 — Additional analysis: association between neighborhood education level and the absolute time of active commuting, N = 537a. (DOCX 12 kb) [file 12889_2017_4464_MOESM2_ESM.docx]

**Additional file 2. Additional analysis: association between neighborhood education level and the absolute time of active commuting, N=537^a^**

|  | RR | 95% CI | RD | 95% CI |
| --- | --- | --- | --- | --- |
| Neighborhood education *(vs. low)* |  |  |  |  |
| *High* | 1.31 | (1.03, 1.66) | 0.27 | (0.03, 0.51) |
| *Middle high* | 1.04 | (0.81, 1.35) | 0.04 | (-0.22, 0.30) |
| *Middle low* | 0.80 | (0.61, 1.04) | -0.23 | (-0.50, 0.04) |

^a^ Negative binomial regression model adjusted for age at the mean, low individual education, living with a child under the age of thirteen, and living in the Rhône “département”..

RR: relative risk; RD: risk difference; CI: confidence interval
