# Supplementary material for: Neighborhood educational disparities in active commuting among women: the effect of distance between the place of residence and the place of work/study (an ACTI-Cités study)
Source: BMC Public Health. 2017 Jun 12;17:569. doi: 10.1186/s12889-017-4464-8 (PMC5469012; doi:10.1186/s12889-017-4464-8)
Supplement: Supplementary file 3 — Sensitivity analysis: association between neighborhood education, distance to work* and the probability of reporting any active commuting (N = 1169). (DOCX 14 kb) [file 12889_2017_4464_MOESM3_ESM.docx]

**Additional file 3. Sensitivity analysis: association between neighborhood education, distance to work* and the probability of reporting any active commuting (N=1169)**

| Regression coefficients | Models 1^a^ | | | | Models 2^b^ |  | | |
| --- | --- | --- | --- | --- | --- | --- | --- | --- |
|  | β | 95% CI |  |  | β | 95% CI |  |  |
| **Neighborhood education** | | | | | | | | |
| *High* | 0.49 | (0.31, 0.66) |  |  | 0.72 | (0.42, 1.02) |  |  |
| *Middle high* | 0.20 | (0.01, 0.40) |  |  | 0.59 | (0.25, 0.93) |  |  |
| *Middle low* | -0.11 | (-0.31, 0.10) |  |  | 0.22 | (-0.37, 0.39) |  |  |
| *Low* | Ref. |  |  |  | Ref. |  |  |  |
| **Distance to work*** |  |  |  |  |  |  |  |  |
| 1 km increase in distance | -0.02 | (-0.03, -0.02) |  |  | -0.01 | (-0.02, 0.01) |  |  |
| **Neighborhood education and distance to work** | | | | | | | | |
| *High x Distance* | - |  |  |  | -0.02 | (-0.04, 0.00) |  |  |
| *Middle high x Distance* | - |  |  |  | -0.03 | (-0.05, -0.01) |  |  |
| *Middle low x Distance* | - |  |  |  | -0.01 | (-0.03, 0.02) |  |  |
| *Low x Distance* | - |  |  |  | Ref. |  |  |  |
| p-value for interaction |  |  |  |  | 0.017 |  |  |  |

^a^ Log-binomial regression model 1 included neighborhood education levels, distance to work, and was adjusted for age at the mean, low individual education, living with a child under the age of thirteen, and living in the Rhône “département”.

^b^ Log-binomial regression model 2 included neighborhood education level, distance to work, the interaction term between neighborhood education levels and distance to work and was adjusted age at the mean, low individual education, living with a child under the age of thirteen, and living in the Rhône “département”.

* Distance to place of work/study was computed based on the travel speed (23km/h for car, 12km/h for public transport, 12km/h for cycling, 4km/h for walking, 10km/h for others active travel modes of transportation).
