# Supplementary material for: Neighborhood educational disparities in active commuting among women: the effect of distance between the place of residence and the place of work/study (an ACTI-Cités study)
Source: BMC Public Health. 2017 Jun 12;17:569. doi: 10.1186/s12889-017-4464-8 (PMC5469012; doi:10.1186/s12889-017-4464-8)
Supplement: Supplementary file 4 — Sensitivity analysis: association between neighborhood education, distance to work* and share of total commuting time spent active among active commuters (N = 537). (DOCX 13 kb) [file 12889_2017_4464_MOESM4_ESM.docx]

**Additional file 4. Sensitivity analysis: association between neighborhood education, distance to work* and share of total commuting time spent active among active commuters (N=537)**

| Regression coefficients | Model 1^a^ |  | Model 2^b^ | |
| --- | --- | --- | --- | --- |
|  | β | 95% CI | β | 95% CI |
| **Neighborhood education** | | | | |
| *High* | 0.18 | (0.06, 0.29) | 0.20 | (0.03, 0.36) |
| *Middle high* | 0.06 | (-0.07, 0.18) | 0.17 | (-0.01, 0.35) |
| *Middle low* | -0.04 | (-0.16, 0.09) | 0.06 | (-0.13, 0.25) |
| *Low* | Ref. |  | Ref. |  |
| **Distance to work*** |  |  |  |  |
| 1km increase in distance | -0.03 | (-0.04, - 0.03) | -0.03 | (-0.04, -0.02) |
| **Neighborhood education and Distance to work** | | | | |
| *High x Distance* | - |  | 0.00 | (-0.01, 0.00) |
| *Middle high x Distance* | - |  | -0.01 | (-0.03, 0.00) |
| *Middle low x Distance* | - |  | -0.01 | (-0.02, 0.00) |
| *Low x Distance* | - |  | Ref. |  |
| p-value for interaction |  |  | 0.198 |  |

^a^ Negative binomial regression model 1 included neighborhood education levels, distance to work, and was adjusted for age at the means, low individual education, living with a child under the age of thirteen, and living in the Rhône “département”.

^b^ ^a^ Negative binomial regression model 2 included neighborhood education level, distance to work, the interaction term between neighborhood education levels and distance to work, and was adjusted for age at the means, low individual education, living with a child under the age of thirteen, and living in the Rhône “département”.

* Distance to place of work/study was computed based on the travel speed (23km/h for car, 12km/h for public transport, 12km/h for cycling, 4km/h for walking, 10km/h for others active travel modes of transportation).
